# Integration of ATAC-Seq and RNA-Seq Identifies Key Genes in Light-Induced Primordia Formation of *Sparassis latifolia*

**DOI:** 10.3390/ijms21010185

**Published:** 2019-12-26

**Authors:** Chi Yang, Lu Ma, Donglai Xiao, Zhenghe Ying, Xiaoling Jiang, Yanquan Lin

**Affiliations:** 1 Institute of Edible Fungi, Fujian Academy of Agricultural Sciences, Fuzhou 350014, China; yc113078@163.com (C.Y.); malujj@163.com (L.M.); xdljiangsu@163.com (D.X.); yingzhenghe@126.com (Z.Y.); gone_to@163.com (X.J.); 2 National and Local Joint Engineering Research Center for Breeding & Cultivation of Featured Edible Fungi, Fujian Academy of Agricultural Sciences, Fuzhou 350014, China

**Keywords:** *Sparassis latifolia*, ATAC-seq, RNA-seq, light, primordia formation

## Abstract

Light is an essential environmental factor for *Sparassis latifolia* primordia formation, but the molecular mechanism is still unclear. In this study, differential expression profiling of light-induced primordia formation (LIPF) was established by integrating the assay for transposase accessible chromatin by sequencing (ATAC-seq) and RNA-seq technology. The integrated results from the ATAC-seq and RNA-seq showed 13 down-regulated genes and 17 up-regulated genes in both the L vs. D and P vs. D groups, for both methods. According to the gene ontology (GO) annotation of these differentially expressed genes (DEGs), the top three biological process categories were cysteine biosynthetic process via cystathionine, vitamin B6 catabolic, and glycine metabolic; the top three molecular function categories were 5-methyltetrahydropteroyltriglutamate-homocysteine S-methyltransferase activity, glycine binding, and pyridoxal phosphate binding; cellular component categories were significantly enriched in the glycine cleavage complex. The KEGG (Kyoto Encyclopedia of Genes and Genomes) enrichment analysis revealed that these genes were associated with vitamin B6 metabolism; selenocompound metabolism; cysteine and methionine metabolism; glycine, serine, and threonine metabolism; and glyoxylate and dicarboxylate metabolism pathways. The expression of most of the DEGs was validated by qRT-PCR. To the best of our knowledge, this study is the first integrative analysis of ATAC-seq and RNA-seq for macro-fungi. These results provided a new perspective on the understanding of key pathways and hub genes in LIPF in *S. latifolia*. It will be helpful in understanding the primary environmental response, and provides new information to the existing models of primordia formation in edible and medicinal fungi.

## 1. Introduction

The transition from mycelium to primordia form, which requires more energy than simple vegetative growth [1], is the most complex and critical developmental event in fruiting body development in various basidiomycete fungi. Understanding the mechanisms of primordia formation has been a goal of research on edible fungi [2]. Recently, the mechanisms of this process have been extensively studied. For instance, the studies on *Pleurotus tuoliensis* [3], *Priurotus eryngii* [4], *Lentinula edodes* [5], *Schizophyllum commune* [6], *Flammulina velutipes* [7], *Ganoderma lucidum* [8], *Cordyceps militaris* [9], *Hypsizygus marmoreus* [10,11], *Termitomyces heimii* [12], *Coprinopsis cinerea* [2,13], and *Pleurotus ostreatus* [14], found some key genes that regulated mushroom development, and demonstrated the underlying molecular mechanisms in this process. However, studies on the mechanisms of fruiting body development in *Sparassis latifolia* are still at a primary stage.

*S. latifolia* was collected from Asian *Sparassis* [15], which has exhibited various biological and pharmacologic activities [16,17,18]. Based on the genome sequence of *S. latifolia* strains SP-C [19], researchers have identified hundreds of differentially expressed proteins during development [20]. Light has previously been shown to be an essential environmental factor for primordia formation in *S. latifolia* [21]. The light response mechanism has also been studied using RNA-Seq [22], and it was found that 157 genes were up-regulated and 171 genes were down-regulated when induced by light. The sequence and expression of several light receptors has also been analyzed [23,24,25], and the *Drosophila*, *Arabidopsis*, *Synechocystis*, and *Homo* species (DASH) type cryptochrome homologous photoreceptor gene *Slcry1* was found to be increased under 300 lx white LED irradiation, with the progress in the developmental stages (mycelia < primordia < fruiting bodies), but *Opsin-1* and *Opsin-2* were down-regulated during the development. However, the molecular mechanism of light-induced primordia formation in *S. latifolia* has not been studied.

In this study, the assay for transposase-accessible chromatin by sequencing (ATAC-seq) [26,27] was used to detect open chromatin regions in light-induced primordia formation (LIPF) in *S. latifolia*. We further identified the key cis-regulatory elements responsible for LIPF in *S. latifolia* signature genes by comparing ATAC-seq results with RNA-seq data. This resulted in the identification of some novel primordia formation inductive genes.

## 2. Results

### 2.1. Results of ATAC-Seq

**Figure 1:**
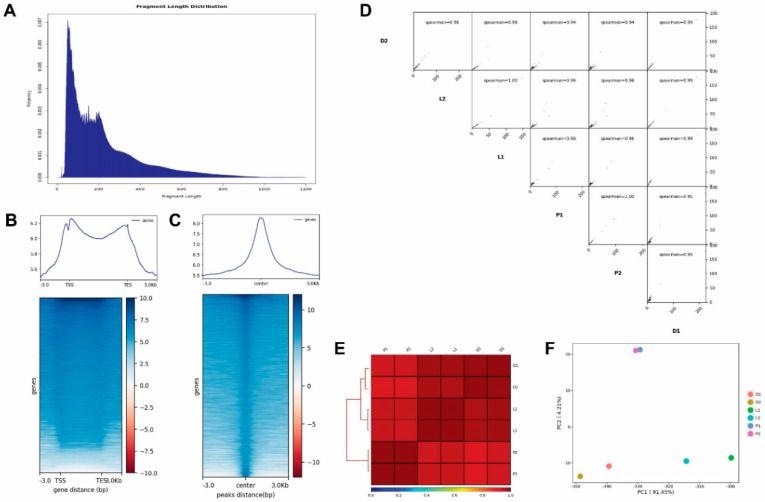
Results of the assay for transposase accessible chromatin by sequencing (ATAC-seq). (**A**) Fragment length distribution map. (**B**,**C**) Mapped reads distributions (from bigwig) across gene bodies and peaks. The X-axis represents the normalized gene or peak length, and the Y-axis represents the read enrichment. The larger the value, the more enriched. TSS stands for the gene start site, and TES stands for the gene stop site. −3.0 represents 3 kb upstream of TSS, and 3.0 kb represents 3 kb downstream of TES. ATAC-seq read distributions are presented as an average plot (up) and heatmap (down). The Deeptools tool was used for this analysis. (**D**) The Pearson correlation results shown by heatmap scatterplot. (**E**) The Pearson correlation results shown by heatmap. (**F**) Principal Components Analysis (PCA) plot.

ATAC-seq was used to detect the landscape of genomic chromatin accessibility during LIPF [27]. After raw data had been obtained from ATAC-seq, the fragment sizes for read pairs, each given a BAM file from paired-end sequencing, were calculated. The expected distribution of fragment lengths was obtained in all ATAC-seq libraries, which possessed both a nucleosome-free fragment and a single-nucleosome fragment, indicating good data quality (Figure 1A). The highest peak on the left of Figure 1A is the nucleosome-free fragment which corresponds to the open chromatin region. Meanwhile, the mononucleosome peak, a cleavage fragment including a nucleosome greater than 147 and less than 147 × 2, was also required to appear in the length distribution map. The results of mapped read distributions across the gene bodies and peaks also showed the good quality of the ATAC-seq (Figure 1B,C). As shown in Table S1, the mappability was found to be above 50%. In total, at least 7723, 10,829, and 8352 high-confidence peaks were identified across all mycelium samples under dark, light, and all primordia samples, respectively.

To further confirm the quality of ATAC-seq, Principal Components Analysis (PCA) and Pearson correlation analysis were performed based on the signals of merged peaks from all samples. The PCA plot sorted the principal components according to the amount of data variability and showed that the samples were clustered by group (Figure 1F). Meanwhile, pair-wise Spearman correlation between any pair of ATAC-seq samples was calculated based on the read counts/signals on merged ATAC-seq peaks from all samples. As shown in Figure 1D,E, the results for the two samples in each group were very close to each other.

### 2.2. Differential Chromatin Accessibility in LIPF

**Figure 2:**
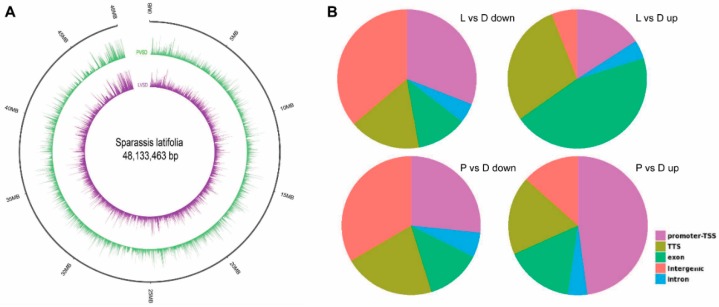
Genomic distribution of differential peaks. (**A**) Circos map of differential peaks. (**B**) The genome-wide distribution of the peaks. The genome-wide functional regions were divided into promoter, downstream TTS, coding exon, intron, and distal intergenic regions.

The differential accessible peaks are shown in Figure 2A and Table S2. There were 1912 down-regulated and 1768 up-regulated peaks in the P vs. D group, while there were only 237 down-regulated and 234 up-regulated peaks in the L vs. D group. The genome-wide functional regions were divided into promoter, downstream gene start site (TTS), coding exon, intron, and distal intergenic regions. The binding sites were annotated, and each binding site could obtain the closest gene to the genome, thereby acquiring a specific distribution of the binding site on the genome. Transcription factors are generally enriched near the start of transcription of the gene, the TSS region. As shown in Figure 2B, the percentages of peaks enriched near the TSS region were 16.60%, 28.76%, 21.33%, and 18.33% in the L vs. D down, L vs. D up, P vs. D down, and P vs. D up groups, respectively. Results also indicated that the percentages of down-regulated and up-regulated peaks enriched near the promoter in the L vs. D group were 36.23% and 15.88%, respectively, while the percentages of down-regulated and up-regulated peaks in the P vs. D group were 26.64% and 47.86%, respectively.

### 2.3. Integration of ATAC-Seq Results with RNA-Seq

**Figure 3:**
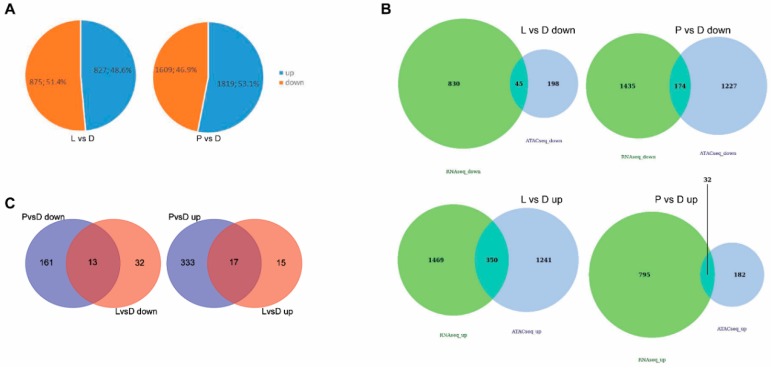
Integration of ATAC-seq and RNA-seq results. (**A**) Statistical pie chart of differentially expressed genes identified using RNA-seq. (**B**) Overlap of differentially expressed genes identified by ATAC-seq and RNA-seq. (**C**) Overlap of differentially expressed genes in the P vs. D and L vs. D groups.

To determine whether the changes in open chromatin regions from the ATAC-seq analysis correlated with the gene expression changes in LIPF, we integrated our ATAC-seq data with RNA-seq data. RNA-Seq was performed using the three cultured group samples. The reads and nucleotides for each sample are shown in Table S3. Based on the differential expression analysis, the light group showed 827 up-regulated genes and 875 down-regulated genes, compared with the dark control group, while the primordia group showed 1819 up-regulated genes and 1609 down-regulated genes, compared with the dark control group (Figure 3A). The integration of ATAC-seq and mRNA-seq results showed the genes that were highly and significantly expressed in each group. After overlapping the results of the two sequence methods, 45 and 174 down-regulated genes were found in the L vs. D and P vs. D groups, respectively, while 32 and 330 up-regulated genes were found in the L vs. D and P vs. D groups, respectively (Figure 3B). With further overlapping of different groups, 30 genes were identified, including 17 up-regulated genes and 13 down-regulated genes (Table S4 and Figure 3C). 

**Figure 4:**
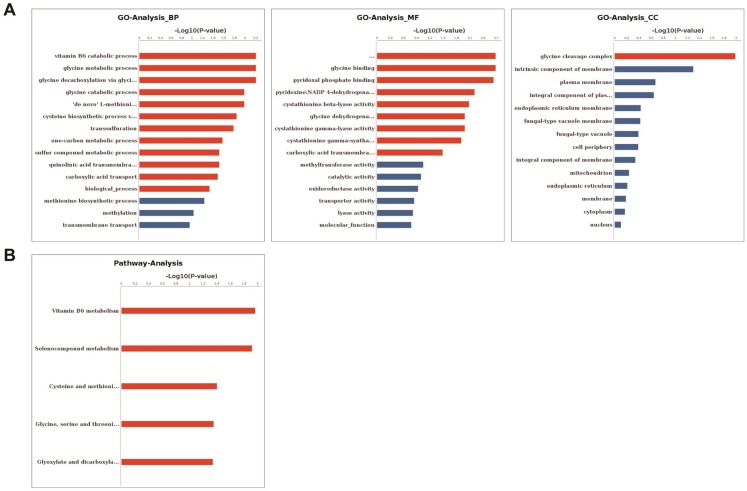
Function analysis of the differentially expressed genes (DEGs). (**A**) Histogram of the gene ontology (GO) classification of the DEGs. Red color represents a significant term (*p* < 0.05), and blue represents a non-significant term. (**B**) KEGG (Kyoto Encyclopedia of Genes and Genomes) annotation for the DEGs.

According to the gene ontology (GO) annotation of these differentially expressed genes (DEGs), the biological process categories included cysteine biosynthetic process via cystathionine, vitamin B6 catabolic, glycine metabolic, glycine catabolic, ‘de novo’ L-methionine biosynthetic process, trans-sulfuration, one-carbon metabolic process, sulfur compound metabolic process, quinolinic acid transmembrane transport, and carboxylic acid transport. In addition, cellular component categories were significantly enriched in the glycine cleavage complex. Similarly, the molecular function categories included 5-methyltetrahydropteroyltriglutamate-homocysteine S-methyltransferase activity, glycine binding, pyridoxal phosphate binding, pyridoxine 4-dehydrogenase activity, cystathionine beta-lyase activity, glycine dehydrogenase (decarboxylating) activity, cystathionine gamma-lyase activity, cystathionine gamma-synthase activity, and carboxylic acid transmembrane transporter activity (Figure 4A and Table S5). It was further noted that the DEGs were significantly assigned to vitamin B6 metabolism; selenocompound metabolism; cysteine and methionine metabolism; glycine, serine, and threonine metabolism; and glyoxylate and dicarboxylate metabolism pathways (Figure 4B).

**Figure 5:**
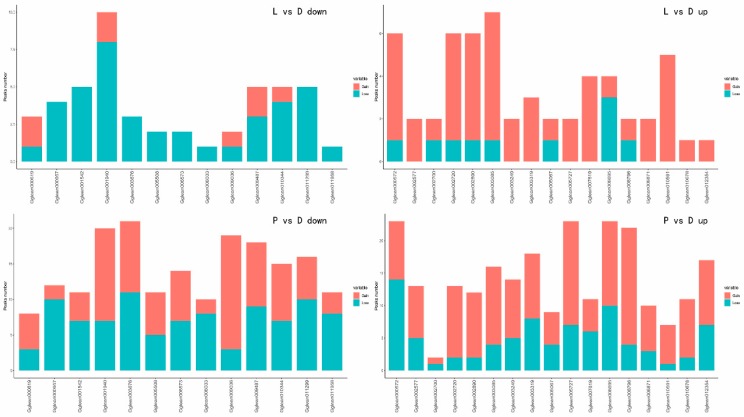
The changes in the chromatin open regions near the DEGs, for each group. The TSS site of the selected gene was expanded upstream and downstream to 100,000 bp, and the differential chromatin open regions of ATAC-seq were analyzed.

In order to more accurately determine the transcription factors that play a regulatory role based on the chromatin open region and further study how transcription factors regulate downstream genes, we analyzed changes in the chromatin open regions upstream and downstream of the DEGs. As Figure 5 shows, most of the signals in the open chromatin region near the down-regulated gene were lost, and the same signals were gained near the up-regulated gene. These results indicated that the change in the regulatory region recognized by ATAC-seq was consistent with the change in the expression of surrounding genes. The genome-wide functional regions of the peaks near these DEGs were annotated. There were 12, 9, 8, 7, and 2 peaks enriched near the promoter, exon, distal intergenic, TTS, and intron regions, respectively (Table S6).

### 2.4. Validation of the Results by qRT-PCR

**Figure 6:**
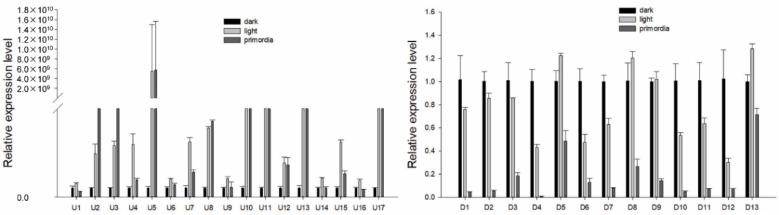
Relative expression levels of the DEGs. X axes are the gene codes of the DEGs, and Y axes are the relative expression levels of the DEGs. Data are presented as mean ± SD.

qRT-PCR was used to quantitatively validate the sequencing data. The DEGs exhibited similar expression patterns to those in the ATAC-seq and RNA-seq experiments (Figure 6), except for U1 (dehydrogenase patE), U9 (SH3 domain-containing protein), U14 (putative methyltransferase-like protein C27D7.08c), U16 (cysteine proteinase 1), D5 (cofactor-independent phosphoglycerate mutase), D8 (meiosis-specific protein hop1), D9 (aldo-keto reductase yakc), and D13 (uncharacterized trans-sulfuration enzyme YHR112C). Therefore, DEGs identified by ATAC-seq and RNA-seq can be further investigated in the future as candidate genes involved in LIPF in *S. latifolia*.

## 3. Discussion

Understanding the molecular mechanisms regulating the fruiting process in macro-fungi, especially in industrially cultivated mushrooms, has long been a goal in mycological research [28]. As a brown rot fungus, *S. latifolia* has exhibited various biological and pharmacological activities [16,17,18]. In China, the total fresh production of *S. latifolia* is over 20 tons/d. However, the cultivation technology is still controlled by a small group of people. Therefore, it is important to elucidate the mechanisms of fruiting body development in *S. latifolia*. Recently, several research groups have sequenced the whole genome sequence of *Sparassis* species [19,29], which will provide base information for further study. Previous study showed that light was an essential environmental factor for the primordia formation in *S. latifolia* [30], but the molecular mechanism of light-induced primordia formation in *S. latifolia* is still unclear.

In this study, we detected the changes in chromatin accessibility and gene expression in the development of mycelia into primordia and combined the treatment of light. We firstly comprehensively analyzed the changes in chromatin accessibility in LIPF in *S. latifolia*. ATAC-seq can identify open chromatin regions that are trimethylated at H3K4, H3K36, and H3K79 [31]. This technology combined with RNA-seq can provide high resolution of the potential functional interactions that occurred during development [32]. ATAC-seq has been widely used in human, plant, and animal research, but few studies have used this technology in fungi research [33,34]. Based on the quality control shown in Figure 1, we think that we successfully applied ATAC-seq to the screening of functional genes of *S. latifolia*, although the number of differentially expressed peaks in the L vs. D group was 507 (Table S2). It was speculated that the possible reason was the time of sample illumination treatment for ATAC-seq. In our previous studies, the expression level of some light receptors was significantly changed after 1 h light treatment [23,24]. So, this time duration was selected for the light treated mycelium group. However, in other studies, light treatment time was usually shorter than 1 h [9,35,36].

The integration of ATAC-seq and RNA-seq results showed that 17 genes were up-regulated and 13 genes were down-regulated in both the L vs. D and P vs. D groups (Table S4 and Figure 3C). The major advantage of this approach is the downstream interactions do not have to be previously known. Integration of ATAC-seq and RNA-seq can determine the transcription factors that play a regulatory role based on the chromatin open region and further study how transcription factors regulate downstream genes. There were 12, 9, 8, 7, and 2 peaks enriched near the promoter, exon, distal intergenic, TTS, and intron regions, respectively (Table S5). The result of GO analysis showed that these DEGs were influenced by a variety of biological processes, particularly metabolic processes (one-carbon metabolic process, sulfur compound metabolic process, glycine metabolic process). When combined with the results of the KEGG pathway analysis of the DEGs, this indicated that functional groups were associated with vitamin B6 metabolism, glycine metabolism, and cystathionine lyase, which might play an important role in LIPF in *S. latifolia*. Metabolism of vitamins was also involved in fruiting body formation in *Lentinula edodes* [37].

Tang et al. found the expression level of *WC-1* was significantly up-regulated in brown mycelia in *L. edodes* [38]. In *C. cinerea*, white collar proteins also play a role in photomorphogenesis and fruiting body development [39]. *WC-1* in *Cordyceps militaris* also could switch the vegetative growth state to primordia differentiation [40]. The expression of *WC-1* in *S. latifolia* was induced by light treatment and up-regulated during development [23]. In this study, the expression of *WC-1* was also up-regulated both in ATAC-seq and RNA-seq, indicating that blue light receptor WC-1 must be associated with light-induced primordium formation in *S. latifolia*. 

Hydrophobins are small proteins of approximately 100 amino acids that are characterized by eight cysteine residues in conserved positions. They are important for the formation and development of fruiting bodies in macro-fungi. In *S. commune*, hydrophobins are involved in the formation of aerial hyphae, as well as the formation of other aerial structures [41]. Hydrophobin 9 in *F. filiformis* was involved in the formation of primordia [42,43]. In this study, a hydrophobin gene, *U10* (Fruiting body protein SC4), was up-regulated in LIPF. 

However, the role of most of these DEGs in primordium formation and stress response in *S. latifolia* is still unclear. Further study is required to investigate the function of these DEGs in LIPF in *S. latifolia*.

## 4. Materials and Methods 

### 4.1. Strains, Culture Conditions, and Isolation of Nucleic Acids

The *S. latifolia* strain SP-C was preserved at the Institute of Edible Fungi, Fujian Academy of Agricultural Sciences (Fuzhou, China). The strain was maintained on potato dextrose agar slants, and seed culture medium was composed of potato (20%), glucose (2%), and fish peptone (0.3%). The pine wood sawdust substrate for *S. latifolia* culture was prepared as described in our previous study [44]. Samples of fungi were divided into three different groups: mycelium group (D), incubated at 23–25 °C in darkness for 28 d after inoculation; light-induced mycelium group (L), 1 h light (200 lx white LED) induced mycelium from group D; and primordia group (P). The D group mycelia were continuously cultured under 14:10 h of light/dark for 2 weeks and primordia were formed. Samples were ground in liquid nitrogen using mortar and pestle. DNA and total RNA were isolated using previous method with some modifications [45].

### 4.2. ATAC-Seq

ATAC-seq was performed as previously described [26]. Raw sequence reads were initially processed for quality control by FastQC (http://www.bioinformatics.babraham.ac.uk/projects/fastqc/) and then Cutadapt was used to remove adapter sequences and poor quality reads [46]. Subsequently, the remaining reads were mapped to the reference genome of *S. latifolia* strain SP-C [19] using BWA (Burrows-Wheeler Alignment) (0.7.10) [47]. SAM files were converted to BAM format using Samtools and used for peak calling. The consensus map was created for each group by merging all samples using the BEDTools [48] merge command. MACS2 (2.1.1) [49] was used to call peaks and an initial threshold was defined as |log2FC| > 0.26, with *p* < 0.05. After performing PCA on the signals of merged peaks from all samples, plotPCA was used to sort the principal components according to the amount of data variability. The pair-wise Spearman correlation between any pair of ATAC-seq samples was calculated based on read counts/signals on merged ATAC-seq peaks from all samples. ATAC-seq peaks were annotated using Homer’s annotatePeaks.pl [50]. Two biological replicates were used.

### 4.3. Integration Analysis of ATAC-Seq and RNA-Seq

The ATAC-seq results were combined with expression data from RNA-seq analysis in order to accurately determine the transcription factors that play a regulatory role based on the chromatin open region and to further study how transcription factors regulated downstream genes. RNA-seq was performed as previously described [5]. Three biological replicates were used for RNA-seq. Raw data (raw reads) of FASTQ format were filtered by Cutadapt [46]. Files were then processed by FASTQC. Reference genomes were directly downloaded from the National Center for Biotechnology Information (NCBI) genome website [19]. The reference genome index was constructed and paired-end clean reads were aligned to the *S. latifolia* genome using STAR [51]. The read numbers mapped to each gene were counted using HTSeq v0.6.1 [52]. DESeq2 R package was used to analyze the differential expression [53]. Genes with |FoldChange| > 1.2 and adjusted *p*-value ≤ 0.05 were assigned as differentially expressed. When several ATAC-seq peaks were associated with one gene, the highest ATAC-seq peak was selected among the gene-proximal peaks. The down-regulated DEGs in RNA-seq were overlapped with the associated gene with a chromatin open region with ATAC-seq signal attenuated, and the up-regulated DEGs in RNA-seq were correlated with ATAC-seq signal-enhanced chromatin open region-related gene. Further, the overlapped DEGs between ATAC-seq and RNA-seq were again overlapped between the L vs. D group and the P vs. D group.

### 4.4. Function Annotation

KEGG pathway analysis was used to discover the significant pathway of differential genes. The significant pathways were selected using Fisher’s exact test, and the *p*-value and false discovery rate (FDR) were used to define the threshold of significance [54]. GO analysis was performed to facilitate the elucidation of the biological implications of unique genes in LIPF [55]. We downloaded the GO annotations from UniProt (http://www.uniprot.org/), NCBI (http://www.ncbi.nlm.nih.gov/), and Gene Ontology (http://www.geneontology.org/). Fisher’s exact test was applied to identify the significant GO categories and FDR was used to correct the *p*-values. 

### 4.5. Gene Expression Analysis

Gene expression analysis was performed by qRT-PCR as previously described [24]. Briefly, total RNA was isolated using TRIzol reagent (Invitrogen, San Diego, CA, USA) and then reverse-transcribed with PrimeScript™ II 1st Strand cDNA Synthesis Kit (Takara, Japan) following the manufacturer’s instructions. cDNA was quantified using SYBR Premix Ex Taq kit (Takara, Japan) on an ABI QuantStudio instrument. Each gene was analyzed in duplicate and normalized to the housekeeping gene *GAPDH* [23]. Primers used in this study are described in Table S2. The reaction mixture contained 4.5 μL cDNA, 0.5 μL primers (10 μM), 12.5 μL 2× SYBR Premix Ex Taq, and ddH_2_O up to 20 μL. The thermal cycling conditions were: 95 °C for 1 min; followed by 40 cycles of 10 s at 95 °C, 34 s at 60 °C, and 60 °C for 1 min. Three biological replicates were used.

## 5. Conclusions

In conclusion, here, we try to use a new technology, integration of ATAC-seq and RNA-seq, to investigate differences in gene expression patterns during primordia formation of *Sparassis latifolia*. We found some key genes that could serve as potential biomarkers to provide insights into the transformation of mycelia to fruiting body in *Sparassis latifolia*.

## Data Availability

The data of ATAC-seq and RNA-seq data in this study have been deposited in NCBI’s Gene Expression Omnibus (GEO) under accession number GSE129983.

## Supplementary Materials

Supplementary materials can be found at https://www.mdpi.com/1422-0067/21/1/185/s1.

## Abbreviations

LIPF	light induced primordia formation
*S. latifolia*	*Sparassis latifolia*
ATAC-seq	assay for transposase accessible chromatin by sequencing
DEPs	differentially expressed peaks
DEGs	differentially expressed genes
PDA	potato dextrose agar
PCA	Principal Components Analysis
GEO	Gene Expression Omnibus
GAPDH	glyceraldehyde-3-phosphate dehydrogenase
qRT-PCR	real-time quantitative PCR
FDR	false discovery rate
