# Supplementary material for: Integration of ATAC-Seq and RNA-Seq Identifies Key Genes in Light-Induced Primordia Formation of Sparassis latifolia
Source: Int J Mol Sci. 2019 Dec 26;21(1):185. doi: 10.3390/ijms21010185 (PMC6981827; doi:10.3390/ijms21010185)
Supplement: Supplementary file 1 [file ijms-21-00185-s001.pdf]

International Journal of Molecular Sciences

Table S1. Summary of the ATAC-seq data for the three groups of *S. latifolia.*

Item	D1	D2	L1	L2	P1	P2
All	1.09×10^{8}	7.00×10^{7}	1.23×10^{8}	1.18×10^{8}	1.27×10^{8}	1.26×10^{8}
UnMapped	2.60×10^{7}	1.52×10^{7}	3.12×10^{7}	2.85×10^{7}	5.54×10^{7}	5.55×10^{7}
Mapped	8.28×10^{7}	5.48×10^{7}	9.18×10^{7}	8.91×10^{7}	7.19×10^{7}	7.01×10^{7}
MappedRate	0.76	0.78	0.75	0.76	0.57	0.56
Unique Mapped	7.80×10^{7}	5.17×10^{7}	8.61×10^{7}	8.38×10^{7}	6.81×10^{7}	6.64×10^{7}
Unique Mapped Rate	0.72	0.74	0.70	0.71	0.54	0.53
Repea tMapped	4.86×10^{6}	3.04×10^{6}	5.74×10^{6}	5.24×10^{6}	3.83×10^{6}	3.75×10^{6}
All Base	1.21×10^{10}	8.40×10^{9}	1.26×10^{10}	1.27×10^{10}	1.39×10^{10}	1.37×10^{10}
UnMapped Base	2.57×10^{9}	1.69×10^{9}	2.96×10^{9}	2.79×10^{9}	5.63×10^{9}	5.64×10^{9}
Mapped Base	9.52×10^{9}	6.71×10^{9}	9.61×10^{9}	9.91×10^{9}	8.25×10^{9}	8.02×10^{9}
Unique Mapped Base	9.04×10^{9}	6.40×10^{9}	9.10×10^{9}	9.41×10^{9}	7.87×10^{9}	7.65×10^{9}
Repeat Mapped Base	4.81×10^{8}	3.18×10^{8}	5.09×10^{8}	4.98×10^{8}	3.80×10^{8}	3.71×10^{8}
Peak Calling	9.40×10^{3}	7.72×10^{3}	1.13×104	1.08×10^{4}	8.51×10^{3}	8.35×10^{3}

Table S2. Differential accessible peaks statistic**s.**

Sample	P vs. D		L vs. D	
	down	up	down	up
PeakNum	1912	1768	273	234

Table S3. Summary of the clean transcriptome sequencing data for the three groups of *S. latifolia***.**

Statistics	D1	D2	D3	L1	L2	L3	P1	P2	P3
All	6.18×10^{7}	7.13×10^{7}	6.54×10^{7}	7.05×10^{7}	6.83×10^{7}	6.10×10^{7}	5.78×10^{7}	6.79×10^{7}	6.40×10^{7}
UnMapped	7.41×10^{6}	8.15×10^{6}	8.07×10^{6}	8.61×10^{6}	9.93×10^{6}	7.93×10^{6}	9.64×10^{6}	1.48×10^{7}	1.01×10^{7}
Mapped	5.44×10^{7}	6.31×10^{7}	5.73×10^{7}	6.19×10^{7}	5.84×10^{7}	5.31×10^{7}	4.81×10^{7}	5.31×10^{7}	5.39×10^{7}
Mapped Rate	0.88	0.89	0.88	0.88	0.86	0.87	0.83	0.78	0.84
Unique Mapped	5.37×10^{7}	6.24×10^{7}	5.66×10^{7}	6.10×10^{7}	5.77×10^{7}	5.24×10^{7}	4.75×10^{7}	5.26×10^{7}	5.31×10^{7}
Unique Mapped Rate	0.87	0.88	0.87	0.87	0.85	0.86	0.82	0.77	0.83
Repeat Mapped	6.87×10^{5}	7.85×10^{5}	6.41×10^{5}	8.66×10^{5}	6.52×10^{5}	6.93×10^{5}	5.66×10^{5}	5.73×10^{5}	7.65×10^{5}
Junction All Mapped	2.21×10^{7}	2.63×10^{7}	2.30×10^{7}	2.45×10^{7}	2.30×10^{7}	2.10×10^{7}	1.91×10^{7}	2.10×10^{7}	2.15×10^{7}
Junction Unique Mapped	2.18×10^{7}	2.59×10^{7}	2.28×10^{7}	2.42×10^{7}	2.27×10^{7}	2.07×10^{7}	1.89×10^{7}	2.08×10^{7}	2.11×10^{7}
All Base	9.27×10^{9}	1.07×10^{10}	9.80×10^{9}	1.06×10^{10}	1.02×10^{10}	9.16×10^{9}	8.66×10^{9}	1.02×10^{10}	9.61×10^{9}
UnMapped Base	1.11×10^{9}	1.22×10^{9}	1.21×10^{9}	1.29×10^{9}	1.49×10^{9}	1.19×10^{9}	1.45×10^{9}	2.22×10^{9}	1.52×10^{9}
MappedBase	8.16×10^{9}	9.47×10^{9}	8.59×10^{9}	9.28×10^{9}	8.76×10^{9}	7.97×10^{9}	7.22×10^{9}	7.97×10^{9}	8.08×10^{9}
Unique Mapped Base	8.05×10^{9}	9.35×10^{9}	8.50×10^{9}	9.15×10^{9}	8.66×10^{9}	7.86×10^{9}	7.13×10^{9}	7.89×10^{9}	7.97×10^{9}
Repeat Mapped Base	1.03×10^{8}	1.18×10^{8}	9.61×10^{7}	1.30×10^{8}	9.78×10^{7}	1.04×10^{8}	8.49×10^{7}	8.59×10^{7}	1.15×10^{8}
InsertSize	679.00	665.00	631.00	760.00	609.00	649.00	645.00	613.00	595.00

Table S4. Summary of the DEGs in integration of ATAC-seq with RNA-seq.

Genes	Code	GenBank No.	Primer pairs （5'→3'）	Descriptions
D1	Gglean000619	MK972849	F:CGCTGTCTGGCCGGTATATT<br>D:AAAGCGAATCCACTGAGGCA	MFS general substrate transporter
D2	Gglean000957	MK972850	F:CCAGGAGCGACCTTGGTATT<br>D:CAAGAACGGGGTATCTCCGA	Delta (12) fatty acid desaturase
D3	Gglean001542	MK972851	F:TCAAGATGCTCGGTGGTGAC<br>D:CAATGAGCATGCACGTCGAG	Putative glycine dehydrogenase
D4	Gglean001940	MK972852	F:GTACGGACACAACCTGCTCA<br>D:CGGATATCCCGGTCCACAAG	Phenol 2-monooxygenase
D5	Gglean003676	MK972853	F:GCCCCCGAGAAGAACATGAA	cofactor-independent phosphoglycerate mutase

			D:CCTCGTCGCCATTGACGATA	
D6	Gglean005508	MK972854	F:ACGCGAGTTAGCTATGGAGC<br>D:CCAAGCAATGACAACGCCAA	Uncharacterized transporter
D7	Gglean006573	MK972855	F:GGTTCCATCCACCATGCAGA<br>D:CTACGCCGGAGATGAAGTCC	Alpha/beta hydrolase
D8	Gglean008033	MK972856	F:AGCACTTTCGTTCGGGTGAT<br>D:CGCTGTCCTGCTTTCCAGTA	Meiosis-specific protein hop1
D9	Gglean009036	MK972857	F:GGATGGCCTCAAGAGGATCG<br>D:CAAGAGGGACTCCATACCGC	Aldo-keto reductase yakc [NADP(+)]
D10	Gglean009487	MK972858	F:AGCTCGAAGAAACTGCACGA<br>D:TCTCGTCCCCAGCCTTGATA	lactate 2-monooxygenase
D11	Gglean010344	MK972859	F:ACTCCAACCTCACGTTCGTC<br>D:CTGATCCGGCGTGAGCTTAT	5-methyltetrahydropteroyltriglutamate--homocysteine methyltransferase
D12	Gglean011299	MK972860	F:TTCGGTAGAGAACCCCCGAT<br>D:CATGTGGGGCTCAACTGCTA	O-methylsterigmatocystin oxidoreductase
D13	Gglean011958	MK972861	F:ACGATTTCGACCCCCTCAAC<br>D:TAACCACCTGTGATGGCGAC	Uncharacterized trans-sulfuration enzyme YHR112C
U1	Gglean000572	MK972862	F:GCCGTCCGACTATGACGAAT<br>D:CGAGTCAGGTATGCGGTCTC	Dehydrogenase patE
U2	Gglean002577	MK972863	F:CGTCACATCGATGAACACGC<br>D:AACCTCGAACGGGAACTCAC	hypothetical protein 1
U3	Gglean002700	MK972864	F:CGTCACATCGATGAACACGC<br>D:AACCTCGAACGGGAACTCAC	Iron-sulfur clusters transporter ATM1
U4	Gglean002720	MK972865	F:TTTCGCTGGGTGCTTATCGT<br>D:GGAACCCTAACTTCACCGCA	hypothetical protein 2
U5	Gglean002890	MK972866	F:AGATGATGAGCGTCTTCCGC<br>D:AACTCGACCATGATGGGCTC	hypothetical protein 3
U6	Gglean003085	MK972867	F:CCAATGCACTGAGACGGACT<br>D:ATGTCTGAAGTGGGAAGCCG	Pre-mRNA-splicing factor CWC22
U7	Gglean003249	MK972868	F:TGCGTCCAAGATATCGTCCG<br>D:GAACAATCGCGATGAGCCAC	hypothetical protein 4
U8	Gglean003319	MK972869	F:TTCACGAGGACCACAAGCTC<br>D:CTGCTTCCCAAACTCGTCCT	Phospholipid methyltransferase
U9	Gglean005067	MK972870	F:CGACGATGATTTGACACCGC<br>D:CTCTCCGGACTGCGATTCTC	SH3 domain-containing protein
U10	Gglean005727	MK972871	F:CCATCACCGTCATCGGAGTT<br>D:AACCAAGTTGCCGTAGCTGT	Fruiting body protein SC4 (Precursor)
U11	Gglean007619	MK972872	F:CCCCTCAGCGTCGATAGTTC<br>D:GAGGTTAGAGGCGACATCGG	Gluconate transport inducer 1
U12	Gglean008695	MK972873	F:CGCGATTCGAAAAGGAGCTG<br>D:AGGATGGAGAGGACGAGCTT	LON peptidase N-terminal domain and RING finger protein
U13	Gglean008796	MK972874	F:TATATGCGCGCAAAGCACAC<br>D:CGAGTAAGATCACCGTCCCG	hypothetical protein 5
U14	Gglean008871	MK972875	F:TGAAGAGCAGACGACTCACG<br>D:ATTTCAGGTTCCGCGATGGA	Putative methyltransferase-like protein C27D7.08c
U15	Gglean010591	KX671998	F:CCAGCATCACCAACTACAAGAA<br>D:CCTCTGCGGGAGTATTATTGAC	White collar 1 protein
U16	Gglean010678	MK972876	F:CCGATACCACCTTCCTCGTG<br>D:AGCCAAGTTCTTGAGGGTCG	Cysteine proteinase 1
U17	Gglean012354	MK972877	F:GTACCACCGGACTCACCTTC<br>D:TACTGCTTTCGCAGACCTGG	Dehydrogenase citC
GAPDH	Gglean007906	MF975750	F:TCATTACCGCACCCTCTTCC<br>D:CCACCACGCCAGTCTTTATG	Glyceraldehyde-3-phosphate dehydrogenase

Table S5. Gene ontology and pathway enrichment analysis of the DEGs.

**Categories**	**Term ID**	**Term**	**Symbol**	**P-Value**	**FDR**	**Enrichment**
biological process	GO:0042820	vitamin B6 catabolic process	Gglean009036	0.006	0.035	160.56
	GO:0006544	glycine metabolic process	Gglean001542	0.006	0.035	160.56

	GO:0019464	glycine decarboxylation via glycine cleavage system	Gglean001542	0.006	0.035	160.56
	GO:0006546	glycine catabolic process	Gglean001542	0.010	0.035	96.33
	GO:0071266	'de novo' L-methionine biosynthetic process	Gglean011958	0.010	0.035	96.33
	GO:0019343	cysteine biosynthetic process via cystathionine	Gglean011958	0.014	0.040	68.81
	GO:0019346	transsulfuration	Gglean011958	0.017	0.040	60.21
	GO:0006730	one-carbon metabolic process	Gglean001542	0.027	0.051	37.05
	GO:0006790	sulfur compound metabolic process	Gglean011958	0.031	0.051	32.11
	GO:1903222	quinolinic acid transmembrane transport	Gglean005508	0.031	0.051	32.11
	GO:0046942	carboxylic acid transport	Gglean005508	0.033	0.051	30.10
	GO:0008150	biological_process	Gglean009036	0.047	0.067	5.47
	GO:0008150	biological_process	Gglean000619	0.047	0.067	5.47
cellular component	GO:0005960	glycine cleavage complex	Gglean001542	0.010	0.145	96.27
molecular function	GO:0003871	5-methyltetrahydropteroyltriglutamate-homocysteine S-methyltransferase activity	Gglean010344	0.002	0.015	480.83
	GO:0016594	glycine binding	Gglean001542	0.002	0.015	480.83
	GO:0030170	pyridoxal phosphate binding	Gglean011958	0.002	0.015	25.99
	GO:0030170	pyridoxal phosphate binding	Gglean001542	0.002	0.015	25.99
	GO:0050236	pyridoxine:NADP 4-dehydrogenase activity	Gglean009036	0.006	0.028	160.28
	GO:0004121	cystathionine beta-lyase activity	Gglean011958	0.008	0.028	120.21
	GO:0004375	glycine dehydrogenase (decarboxylating) activity	Gglean001542	0.010	0.028	96.17
	GO:0004123	cystathionine gamma-lyase activity	Gglean011958	0.010	0.028	96.17
	GO:0003962	cystathionine gamma-synthase activity	Gglean011958	0.012	0.030	80.14
	GO:0046943	carboxylic acid transmembrane transporter activity	Gglean005508	0.033	0.069	30.05
KEGG	PATH:00750	Vitamin B6 metabolism	Gglean009036	0.011	0.030	91.37
	PATH:00450	Selenocompound metabolism	Gglean010344	0.012	0.030	82.23
	PATH:00270	Cysteine and methionine metabolism	Gglean010344	0.040	0.046	24.92
	PATH:00260	Glycine, serine and threonine metabolism	Gglean001542	0.044	0.046	22.23
	PATH:00630	Glyoxylate and dicarboxylate metabolism	Gglean001542	0.046	0.046	21.64

Table S6. Information of peaks enriched near these DEGs.

Gene	PeakID	Peak Score	Annotation	Distance to TSS	Nearest PromoterID
D1	peaks616	4.17	TTS	2340	Gglean000619
D2	peaks688	11.11	promoter	-646	Gglean000957
D3	peaks4313	2.86	exon	2542	Gglean001542
D3	peaks4312	10.82	promoter	-185	Gglean001542
D4	peaks3981	6.07	TTS	744	Gglean001940

D5	peaks5312	6.87	promoter	-785	Gglean003676
D6	peaks6386	9.21	promoter	-871	Gglean005508
D7	peaks6891	5.41	promoter	-276	Gglean006573
D8	peaks2007	21.15	exon	842	Gglean008033
D9	peaks8706	15.25	promoter	-161	Gglean009036
D10	peaks2432	15.54	exon	741	Gglean009487
D11	peaks2601	17.31	TTS	2444	Gglean010344
D12	peaks10056	19.80	promoter	-871	Gglean011299
D13	peaks10409	317.64	Intergenic	-32526	Gglean011958
D13	peaks10408	68.56	Intergenic	-26507	Gglean011958
D13	peaks10406	3.06	Intergenic	-8245	Gglean011958
D13	peaks10405	2.79	Intergenic	-7116	Gglean011958
D13	peaks10407	2.53	Intergenic	-25360	Gglean011958
U1	peaks519	4.08	exon	2648	Gglean000572
U2	peaks5074	6.03	intron	376	Gglean002577
U3	peaks12468	11.23	exon	755	Gglean002700
U4	peaks5484	6.92	TTS	462	Gglean002720
U4	peaks5485	4.07	Intergenic	-2537	Gglean002720
U5	peaks5486	16.79	Intergenic	-1301	Gglean002890
U6	peaks5650	13.42	exon	758	Gglean003085
U6	peaks5649	2.54	exon	212	Gglean003085
U7	peaks5701	2.32	TTS	2161	Gglean003249
U8	peaks5820	2.52	intron	2154	Gglean003319
U9	peaks7234	7.79	exon	484	Gglean005067
U10	peaks7634	8.92	promoter	-822	Gglean005727
U11	peaks9215	9.63	Intergenic	-1149	Gglean007619
U12	peaks9759	9.20	promoter	-794	Gglean008695
U13	peaks9814	17.10	promoter	-171	Gglean008796
U14	peaks9893	5.76	promoter	-685	Gglean008871
U15	peaks11140	2.81	exon	1830	Gglean010591
U15	peaks11141	15.52	promoter	-340	Gglean010591
U16	peaks11214	2.67	TTS	365	Gglean010678
U17	peaks4041	2.52	TTS	-2047	Gglean012354
